# Supplementary material for: Simulation and quantitative analysis of spatial centromere distribution patterns
Source: bioRxiv. 2025 Jan 24:2025.01.22.634320. Preprint. [Version 1] doi: 10.1101/2025.01.22.634320 (PMC11785228; doi:10.1101/2025.01.22.634320)
Supplement: Supplement 1 [file NIHPP2025.01.22.634320v1-supplement-1.pdf]

bioRxiv preprint doi: https://doi.org/10.1101/2025.01.22.634320; this version posted January 24, 2025. The copyright holder for this preprint (which was not certified by peer review) is the author/funder, who has granted bioRxiv a license to display the preprint in perpetuity. It is made available under aCC-BY 4.0 International license.

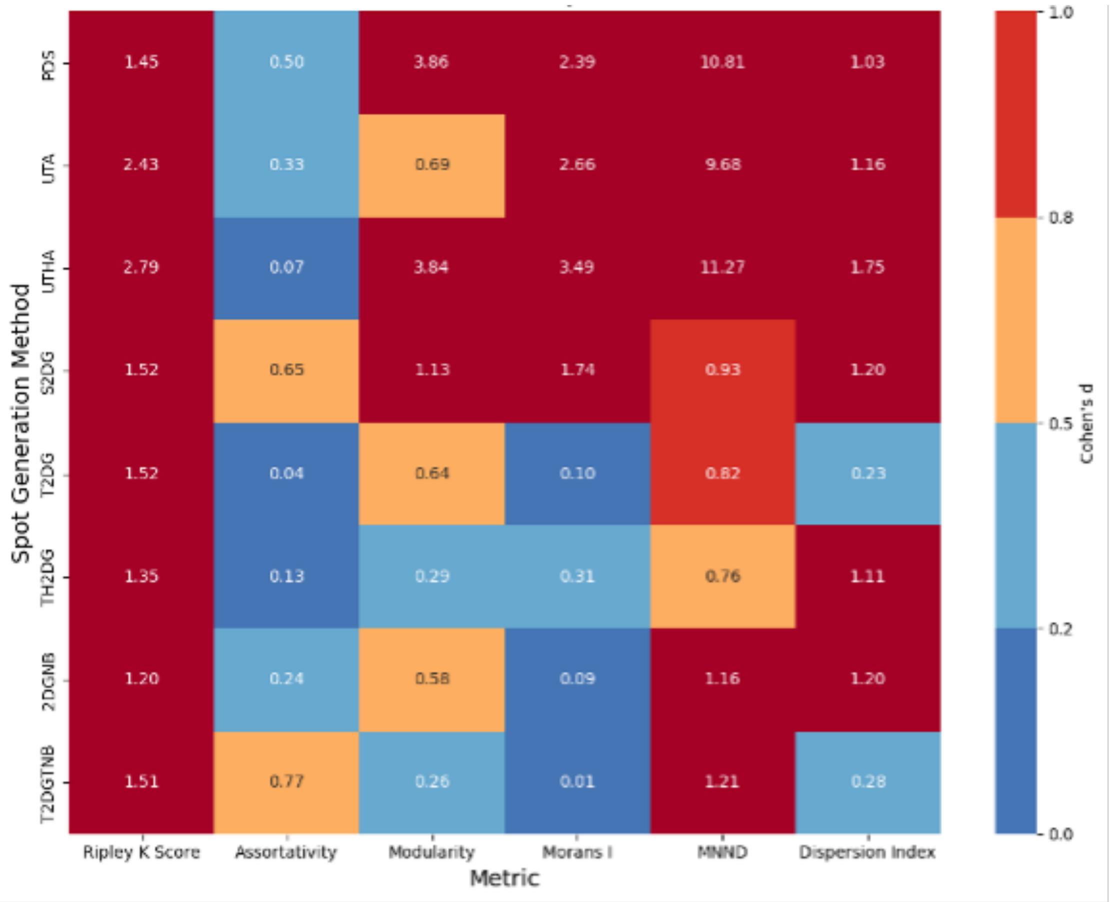

Supplementary Figure 1. Heatmap displaying Cohen's D values for clustering metrics across synthetic spatial distribution models. Metrics include Ripley's K Score, Assortativity, Modularity, Moran's I, Mean Nearest Neighbor Distance (MNND), and Dispersion Index. Cohen's D values quantify the effect size between CSR and other spatial distributions: negligible (D<0.2, dark blue), small (0.2<D < 0.5, light blue), medium (0.5 ≤ D < 0.8, orange), and large (D ≥ 0.8, red). Ripley's K Score consistently demonstrates large effect sizes across most spatial distribution models, indicating substantial differences from CSR. MNND also shows significant differences but is sensitive to dispersion in certain models (e.g., PDS). Other metrics, such as Assortativity and Moran's I, exhibit moderate to small effect sizes for specific distributions, highlighting their limitations in detecting clustering changes robustly. These results support Ripley's K Score as a reliable metric for distinguishing centromere clustering patterns.

bioRxiv preprint doi: https://doi.org/10.1101/2025.01.22.634320; this version posted January 24, 2025. The copyright holder for this preprint (which was not certified by peer review) is the author/funder, who has granted bioRxiv a license to display the preprint in perpetuity. It is made available under aCC-BY 4.0 International license.

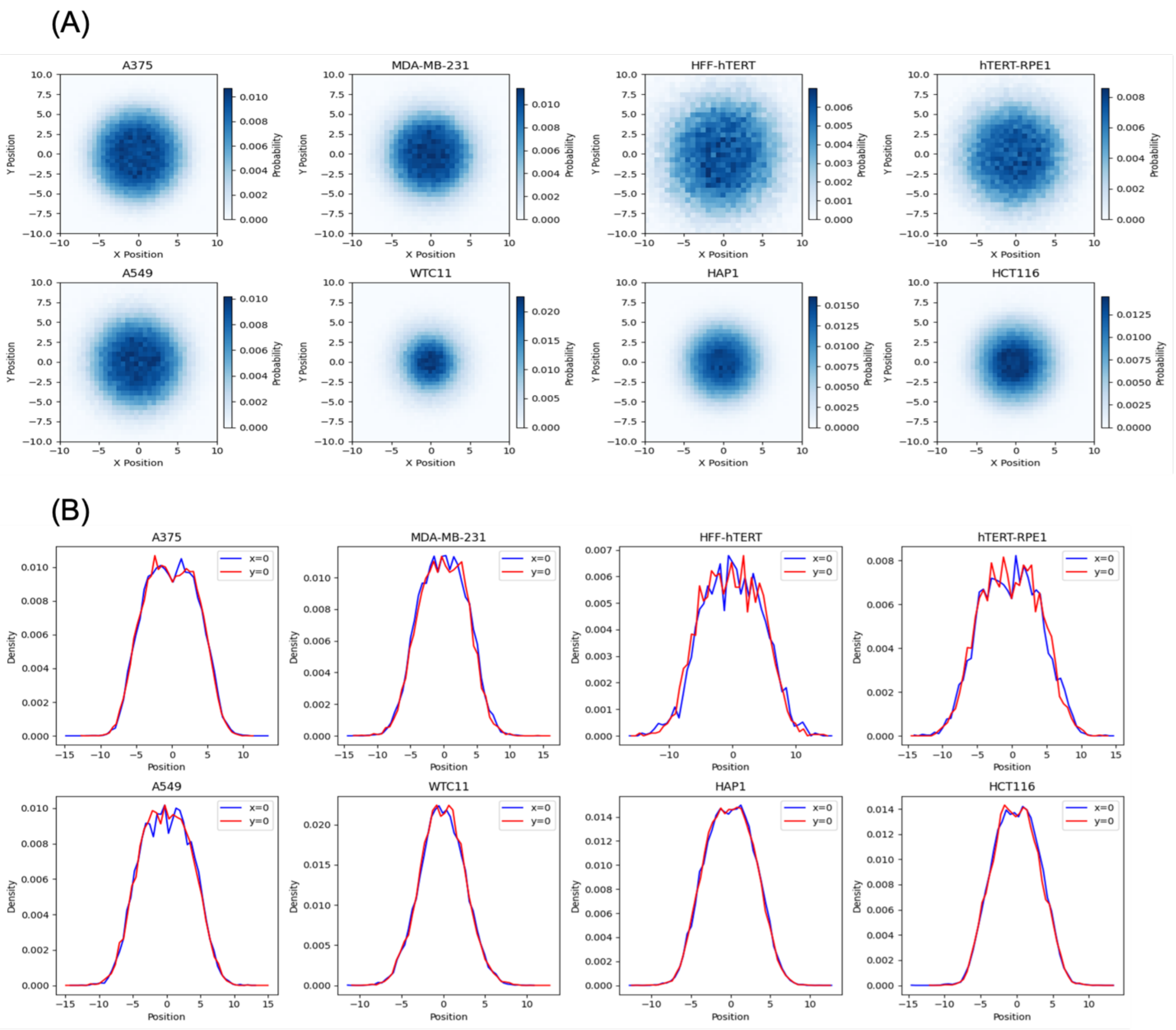

Supplementary Figure 2. Spatial distribution of standardized centromere locations across eight wild-type cell lines. (A) Overlay of nucleus-centered spot locations from HCT116 (colon), A375 (melanoma), MDA-MB-231 (breast), HFF-hTERT (fibroblast), hTERT-RPE1 (retinal), A549 (lung), HAP1 (myeloid), and WTC11 (embryonic stem cells) (A) 2D histogram showing the distribution of CENP-C spots relative to nuclear center (0,0), revealing a doughnut-shaped pattern in all cell lines. (B) Line plot analysis at x=0 and y=0 demonstrating lower spot density at the nuclear center and higher density between center and nuclear edge.

bioRxiv preprint doi: https://doi.org/10.1101/2025.01.22.634320; this version posted January 24, 2025. The copyright holder for this preprint (which was not certified by peer review) is the author/funder, who has granted bioRxiv a license to display the preprint in perpetuity. It is made available under aCC-BY 4.0 International license.

Supplementary Table 1. The sources of cell lines, culture conditions, media compositions and relevant references of respective culture protocols for the eight cell lines.

Metric	*Reference Group*	*Comparison Group*	*Stat*	*p-value*	*Corrected p-value*	*Significant*
Ripley's K Score	CSR	UTHA	19621	4.37E-303	**5.8254e-303**	TRUE
Ripley's K Score	CSR	2DGNB	625446	0	**0.0000e+00**	TRUE
Ripley's K Score	CSR	PDS	930000	5.86E-298	**6.6992e-298**	TRUE
Ripley's K Score	CSR	UTA	33428	4.85E-286	**4.8478e-286**	TRUE
Ripley's K Score	CSR	T2DGTNB	893178	0	**0.0000e+00**	TRUE
Ripley's K Score	CSR	S2DG	525861.5	0	**0.0000e+00**	TRUE
Ripley's K Score	CSR	T2DG	1055834	0	**0.0000e+00**	TRUE
Ripley's K Score	CSR	TH2DG	1206212.5	0	**0.0000e+00**	TRUE
Assortativity	CSR	UTHA	536942	0.00422625	**4.8300e-03**	TRUE
Assortativity	CSR	2DGNB	4287014	9.58E-14	**1.9162e-13**	TRUE
Assortativity	CSR	PDS	622797	1.92E-21	**5.1138e-21**	TRUE
Assortativity	CSR	UTA	593698	3.99E-13	**6.3810e-13**	TRUE
Assortativity	CSR	T2DGTNB	2756891	2.25E-121	**1.7962e-120**	TRUE
Assortativity	CSR	S2DG	3160118	2.72E-82	**1.0865e-81**	TRUE
Assortativity	CSR	T2DG	4934849	0.49622343	4.96E-01	FALSE
Assortativity	CSR	TH2DG	4542913	1.81E-06	**2.4093e-06**	TRUE
Modularity	CSR	UTHA	5385	0	**0.0000e+00**	TRUE
Modularity	CSR	2DGNB	3349885	1.47E-66	**2.3572e-66**	TRUE
Modularity	CSR	PDS	997501	0	**0.0000e+00**	TRUE
Modularity	CSR	UTA	304291	6.94E-52	**9.2591e-52**	TRUE
Modularity	CSR	T2DGTNB	4236213	1.50E-15	**1.4978e-15**	TRUE
Modularity	CSR	S2DG	8020951	1.72E-218	**4.5798e-218**	TRUE
Modularity	CSR	T2DG	6760547	1.66E-75	**3.3104e-75**	TRUE
Modularity	CSR	TH2DG	6028129	6.75E-27	**7.7162e-27**	TRUE
Moran's I	CSR	UTHA	7523	0	**0.0000e+00**	TRUE
Moran's I	CSR	2DGNB	4882722	0.22062501	2.21E-01	FALSE
Moran's I	CSR	PDS	972447	4.71E-293	**9.4114e-293**	TRUE
Moran's I	CSR	UTA	21241	7.05E-301	**1.8803e-300**	TRUE
Moran's I	CSR	T2DGTNB	5407525	2.08E-05	**2.3757e-05**	TRUE
Moran's I	CSR	S2DG	9168219	0	**0.0000e+00**	TRUE
Moran's I	CSR	T2DG	5543492	1.38E-08	**1.8349e-08**	TRUE
Moran's I	CSR	TH2DG	6712341	1.57E-71	**2.5143e-71**	TRUE
MNND	CSR	UTHA	1000000	0	**0.0000e+00**	TRUE
MNND	CSR	2DGNB	8778986	0	**0.0000e+00**	TRUE
MNND	CSR	PDS	0	0	**0.0000e+00**	TRUE
MNND	CSR	UTA	1000000	0	**0.0000e+00**	TRUE
MNND	CSR	T2DGTNB	8609522	0	**0.0000e+00**	TRUE
MNND	CSR	S2DG	8118659	1.03E-232	**1.3698e-232**	TRUE
MNND	CSR	T2DG	7673964	1.24E-171	**1.4190e-171**	TRUE
MNND	CSR	TH2DG	7388535	2.34E-137	**2.3425e-137**	TRUE
Dispersion Index	CSR	UTHA	93180	7.60E-218	**1.5205e-217**	TRUE
Dispersion Index	CSR	2DGNB	9094784	0	**0.0000e+00**	TRUE
Dispersion Index	CSR	PDS	764597	2.62E-93	**3.4939e-93**	TRUE
Dispersion Index	CSR	UTA	200042	2.33E-119	**3.7284e-119**	TRUE
Dispersion Index	CSR	T2DGTNB	4461777	1.90E-08	**2.1661e-08**	TRUE
Dispersion Index	CSR	S2DG	9583300	0	**0.0000e+00**	TRUE
Dispersion Index	CSR	T2DG	5230485	0.01607436	**1.6074e-02**	TRUE
Dispersion Index	CSR	TH2DG	8142653	2.82E-236	**7.5134e-236**	TRUE

bioRxiv preprint doi: https://doi.org/10.1101/2025.01.22.634320; this version posted January 24, 2025. The copyright holder for this preprint (which was not certified by peer review) is the author/funder, who has granted bioRxiv a license to display the preprint in perpetuity. It is made available under aCC-BY 4.0 International license.

Supplementary Table 2. Statistical testing results for pairwise Mann-Whitney U tests, corrected using the Benjamini-Hochberg False Discovery Rate (BH-FDR) method, comparing clustering metrics calculated for CSR and all other spot generation methods.

Metric	*Distribution*	*Percent Change (%)*
Ripley's K Score	CSR	59.5567608
Ripley's K Score	PDS	inf
Ripley's K Score	UTA	12.0172256
Ripley's K Score	UTHA	3.14291737
Ripley's K Score	S2DG	2.83261342
Ripley's K Score	T2DG	1.36536147
Ripley's K Score	TH2DG	1.86411296
Ripley's K Score	2DGNB	1.59446813
Ripley's K Score	T2DGTNB	4.94364368
Assortativity	CSR	682.231385
Assortativity	PDS	49726.8791
Assortativity	UTA	44496.7239
Assortativity	UTHA	857.562303
Assortativity	S2DG	339.190031
Assortativity	T2DG	683.523035
Assortativity	TH2DG	554.275336
Assortativity	2DGNB	406.583787
Assortativity	T2DGTNB	280.373907
Modularity	CSR	52.4011886
Modularity	PDS	54.152431
Modularity	UTA	100
Modularity	UTHA	39.8715057
Modularity	S2DG	55.0145623
Modularity	T2DG	50.1452432
Modularity	TH2DG	52.453178
Modularity	2DGNB	50.1102691
Modularity	T2DGTNB	51.4916329
Moran's I	CSR	17.7412248
Moran's I	PDS	17.6846799
Moran's I	UTA	132.437197
Moran's I	UTHA	13.5630049
Moran's I	S2DG	15.6657324
Moran's I	T2DG	14.8906965
Moran's I	TH2DG	15.3107854
Moran's I	2DGNB	14.7192826
Moran's I	T2DGTNB	15.4520555
MNND	CSR	77.2792286
MNND	PDS	32.7068625
MNND	UTA	1160.51333
MNND	UTHA	273.062842
MNND	S2DG	69.7615691
MNND	T2DG	72.571224
MNND	TH2DG	72.5743327
MNND	2DGNB	75.9056292
MNND	T2DGTNB	84.1758623
Dispersion Index	CSR	2.34686669
Dispersion Index	PDS	2.25438507
Dispersion Index	UTA	38.722864
Dispersion Index	UTHA	2.30607869
Dispersion Index	S2DG	3.97306092
Dispersion Index	T2DG	2.79085127
Dispersion Index	TH2DG	2.862863
Dispersion Index	2DGNB	2.36469957
Dispersion Index	T2DGTNB	3.02788452

bioRxiv preprint doi: https://doi.org/10.1101/2025.01.22.634320; this version posted January 24, 2025. The copyright holder for this preprint (which was not certified by peer review) is the author/funder, who has granted bioRxiv a license to display the preprint in perpetuity. It is made available under aCC-BY 4.0 International license.

Supplementary Table 3. The percent change calculated as average value for each metric for up to 30 spots removed from the initial 46 spots compared to the value with 46 spots.

	CULTURE CONDITIONS				MEDIA COMPOSITION					
**CELL LINE**	**Split ratio**	**Temperature**	**Percent CO2**	**Percent O2**	**Medium**	**Serum**	**Substrate**	**Supplement**	**Source**	**Reference/ protocol**
WTC11	1:8	37°C	5%	Ambient	mTESR1 (STEMCELL, # 85850)	none	matrigel (Corning, #356231)	Penicillin-Streptomycin (Gibco, #15140122)	Coriell Cell Repository (#GM25256)	https://www.allencell.org/written-protocols.html
HFF-HTERT	1:8	37°C	5%	Ambient	DMEM: high glucose, GlutaMAX Supplement, pyruvate (Fisher Scientific, #10569010)	10% FBS (Gibco, #10082147)	none	Penicillin-Streptomycin (Gibco, #15140122)	Job Dekker (RRID: CVCL_VC40)	https://data.4dnucleome.org/protocols/84ee9070-e0c0-478f-9bec-7df22f3e89c2/
HTERT-RPE1 CAS9	1:8	37°C	5%	Ambient	DMEM high glucose pyruvate (Gibco, #11995065)	10% FBS (Gibco, #10082147)	none	Penicillin-Streptomycin (Gibco, #15140122)	Thomas Gonatopoulos-Pournatzis laboratory	Hart et al., Cell, 2015 PMID: 26627737
HAP1 CAS9	1:8	37°C	5%	Ambient	DMEM high glucose pyruvate (Gibco, #11995065)	10% FBS (Gibco, #10082147)	none	Penicillin-Streptomycin (Gibco, #15140122)		Horizon Discovery (HD Cas9-011)
A375 CAS9	1:8	37°C	5%	Ambient	DMEM high glucose pyruvate (Gibco, #11995065)	10% FBS (Gibco, #10082147)	none	Penicillin-Streptomycin (Gibco, #15140122)		Horizon Discovery (HD Cas9-001)
HCT116 CAS9	1:8	37°C	5%	Ambient	RPMI-1640 (ATCC, #30-2001)	10% FBS (Gibco, #10082147)	none	Penicillin-Streptomycin (Gibco, #15140122)		Hart et al., Cell, 2015 PMID: 26627737
A549 CAS9	1:8	37°C	5%	Ambient	RPMI-1640 (ATCC, #30-2001)	10% FBS (Gibco, #10082147)	none	Penicillin-Streptomycin (Gibco, #15140122)		Hart et al., Cell, 2015 PMID: 26627737
MDA-MB-231 CAS9	1:8	37°C	5%	Ambient	RPMI-1640 (ATCC, #30-2001)	10% FBS (Gibco, #10082147)	none	Penicillin-Streptomycin (Gibco, #15140122)		Horizon Discovery (HD Cas9-014)
